# Molecular analysis of intracanal microbial reduction to compare the effectiveness of glycyrrhizin and calcium hydroxide as intracanal medications: a randomised controlled trial

**DOI:** 10.1186/s12903-026-07906-6

**Published:** 2026-03-11

**Authors:** Omar Talib Khaleefah, Ahmed M. El-Baz, Mohamed Ahmed Gomaa, Amany Elsaid Badr

**Affiliations:** 1 Department of Endodontic, Faculty of Dentistry, Mansoura University, Mansoura, 35516 Egypt; 2 Department of Microbiology and Immunology, Faculty of Pharmacy, Delta University for Science and Technology, Gamasa, 11152 Egypt; 3 Department of Nursing, College of Alghad Applied Medical Sciences, Qassim, Saudi Arabia

**Keywords:** Apical periodontitis, DNA, Herbal extracts, RNA, Root canal infection

## Abstract

**Background:**

Endodontic treatment aims to thoroughly clean and disinfect the root canal system; however, current techniques, instruments, and irrigants limit complete root canal sterilisation. This study aimed to assess and compare the antibacterial effectiveness of glycyrrhizin (Gly) and calcium hydroxide (Ca(OH)_2_) as intracanal medicaments using molecular analysis.

**Methods:**

This double-blind (participant- and assessor-blind) study included 28 patients with single-rooted teeth diagnosed with pulp necrosis and asymptomatic apical periodontitis. Following field disinfection, bacterial samples were collected before and after chemomechanical preparation (S1 and S2, respectively) and 7 days after intracanal medicament placement (S3). Participants were randomly assigned to Group 1 (Ca(OH)_2_) or Group 2 (Gly) (*n* = 14 each). All samples were promptly transferred for molecular analysis, and bacterial DNA and RNA levels were assessed using real-time polymerase chain reaction. Data were log-transformed and evaluated using mixed‑effects two‑way repeated‑measures models (time × treatment). The change in log‑transformed DNA and RNA between S2 and S3 (Δlog S3–S2) was compared between groups using the Mann–Whitney U test, with a significance level of 0.05.

**Results:**

Twenty-eight participants (14 per group) were included in the statistical analysis at S1, S2. At S3, data were available for 10 and 11 participants for DNA and 11 and 10 participants for RNA in groups 1 and 2, respectively. Mixed‑effects two‑way repeated‑measures analysis showed significant effects of group and time on log‑transformed bacterial DNA and RNA levels, with a significant group×time interaction (*p* < 0.0001). The reduction in log‑transformed RNA (primary end-point) and DNA from S2 to S3 (Δlog S3–S2) was significantly greater in Group 2 than in Group 1 (RNA: *p* < 0.0001; DNA: *p* = 0.0004).

**Conclusions:**

Compared with Ca(OH)_2_, Gly demonstrated greater antibacterial activity through greater reductions in bacterial DNA and RNA levels. This may support the potential of Gly as an alternative intracanal medicament during root canal therapy.

**Trial registration:**

This study was retrospectively registered on clinicaltrials.gov under the identifier NCT06291623 on March 4, 2024.

## Background

Pulpal and periapical diseases mainly arise from bacterial infection and its toxic by-products. The primary goal of endodontic treatment is to thoroughly clean and disinfect the root canal system while preventing reinfection in the root canals and surrounding periapical tissues [1, 2]. However, current techniques, instruments, and irrigants limit the complete sterilisation of root canals. Therefore, the emphasis should be on reducing bacterial counts within the canals to levels that support the healing of periapical tissues [3]. In such cases, additional disinfection steps involving intracanal dressings may be required [4].

Calcium hydroxide (Ca(OH)_2_) is widely recognised as the most frequently employed intracanal medicament in endodontics, owing to its antibacterial efficacy against most root canal bacteria, high pH, biocompatibility, and ability to promote healing. However, it is ineffective against certain microbes, such as *Enterococcus faecalis* [4].

Nowadays, herbal extracts are increasingly used in medical and dental practice owing to their biocompatibility, anti-microbial activity, and anti-inflammatory and anti-oxidant properties. Glycyrrhizin (Gly), the principal active component of liquorice root, exhibits anti-microbial effects by modulating gene expression, inhibiting bacterial growth, and reducing microbial toxin production. Badr et al. [5] reported that a liquorice extract containing 7.5% Gly (> 95% purity), either alone or combined with Ca(OH)_2_, displayed potent bactericidal action against *E. faecalis*. Furthermore, it demonstrated compatibility with fibroblasts in tissue culture. These findings suggest that Gly could represent a valuable alternative to Ca(OH)_2_ as a root canal medicament.

Recently, Eltantawi et al. [6] conducted a randomised clinical trial using microbiological culture methods and showed that Gly gel significantly reduced bacterial load in necrotic root canals. Its antibacterial efficacy was comparable to that of Ledermix and superior to that of Ca(OH)_2_. These results further support the potential of Gly as a promising herbal intracanal medicament.

Intracanal bacterial levels and species before and after chemomechanical preparation have been extensively investigated using both culture-based (phenotypic) and molecular (genotypic) methods [3]. Owing to their high sensitivity and ability to detect bacteria that are difficult to culture or have not yet been cultivated, molecular techniques have become the preferred method for evaluating persistent endodontic bacteria [7].

Approximately 40%–60% of bacterial taxa in endodontic infections remain uncultivated and are not phenotypically characterised. These uncultivated taxa can be just as pathogenic as their cultivable counterparts, and many of them are associated with apical periodontitis [8]. Molecular microbiology has corroborated and expanded upon culture-based findings, thereby enhancing the understanding of endodontic infections [9].

Notably, DNA alone may not be a highly reliable marker to target viable bacteria, as non-viable bacteria can still retain their DNA. In contrast, RNA is more closely associated with cellular metabolism and gene expression; thus, it is a more accurate indicator of bacterial viability [10]. Therefore, evaluation of both DNA and RNA using real-time polymerase chain reaction offers a more comprehensive understanding by providing information on both bacterial levels and their metabolic activity [10].

Clinical studies employing molecular-based techniques to assess the anti-microbial efficacy of Ca(OH)_2_ as an intracanal medicament have reported mixed or conflicting results [8, 11–13]. Some researchers have reported that bacterial counts following the use of inter-appointment medication were reduced compared with those after chemomechanical preparation [11, 13]. However, others have found no significant differences before and after using intracanal medicaments [8, 12].

To the best of our knowledge, no molecular data regarding the antibacterial effectiveness of Gly as an intracanal medicament in clinical practice are currently available. Therefore, this study aimed to compare the antibacterial efficacy of Gly gel and Ca(OH)_2_ as intracanal medicaments in patients with pulp necrosis and asymptomatic apical periodontitis using molecular analysis of bacterial DNA and RNA. Building on the findings of previous in vitro studies using liquorice extracts and culture‑based clinical studies involving Gly [5, 6], the present study is the first to evaluate Gly as an intracanal medicament using RNA‑based molecular methods, providing novel information on viable bacterial loads and bacterial viability in the root canal.

## Methods

### Study design and ethical approval

**Fig. 1:**
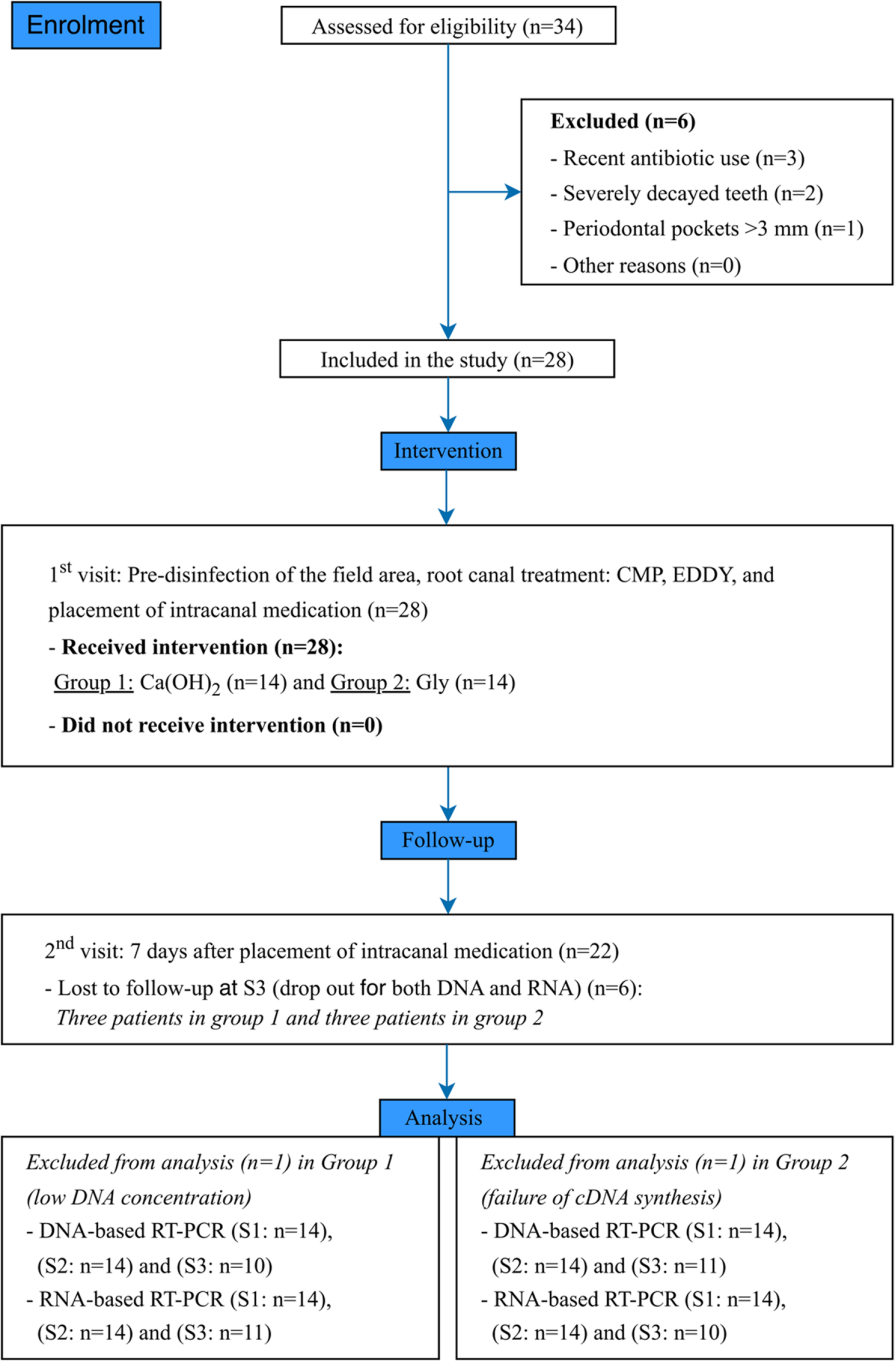
CONSORT flow diagram. RT-PCR, real-time polymerase chain reaction; Ca(OH)_2_, calcium hydroxide; Gly, glycyrrhizin

The study was conducted in accordance with the CONSORT (Consolidated Standards of Reporting Trials) statement for Randomised Trials guidelines (Fig. 1) [14] as a parallel, superiority, double-blind (participant- and assessor-blind), randomised active-controlled trial, and was retrospectively registered on clinicaltrials.gov under the identifier NCT06291623 on March 4, 2024. Ethical approval was obtained from the Research Ethics Committee of the Faculty of Dentistry, Mansoura University (approval number A01050422). Written informed consent was obtained from all participants prior to enrolment.

### Sample size calculation

The sample size was calculated using G*Power software (version 3.1.9.7; Heinrich Heine University, Düsseldorf, Germany) according to the findings of an initial pilot study. The analysis assumed a two-tailed significance level of α = 0.05 with a statistical power of 85%. The results indicated that 10 participants per group would be sufficient to detect a statistically significant difference between the two medicaments. To compensate for potential dropouts, 14 patients were included in each group, yielding a total sample size of 28 participants.

### Patient selection

Inclusion criteria comprised single-rooted teeth with a single canal, pulp necrosis (no response to sensitivity testing), asymptomatic apical periodontitis, and age between 19 and 60 years. Exclusion criteria included teeth presenting with symptoms (pain, tenderness to percussion, or swelling), incompletely developed roots, antibiotic therapy within the preceding 3 months, systemic disease, inability to achieve rubber dam isolation, periodontal pockets deeper than 3 mm, or the presence of root fractures and severely decayed teeth.

A total of 34 patients were assessed for eligibility in the Endodontic Department outpatient clinic at the Faculty of Dentistry, Mansoura University. Six were excluded for not meeting the inclusion criteria [recent antibiotic use (*n* = 3); severely decayed teeth (*n* = 2) and periodontal pockets > 3 mm (*n* = 1)]. Consequently, 28 patients who fulfilled the eligibility criteria were were enrolled and randomized (14 per group). Demographic and clinical baseline characteristics (e.g., age, sex, tooth type, and root canal preparation) were recorded for all enrolled participants and are summarized in Table 1.

**Table 1:** Demographic and clinical characteristics of the participants

		*N*	%	Ca(OH)_2_	Gly
Age	19–40	10	36	5	5
	> 40	18	64	9	9
Sex	Male	9	33	4	5
	Female	19	67	10	9
Tooth type	Maxillary incisors	25	89	12	13
	Maxillary canine	3	11	2	1
Root canal preparation	WaveOne Gold, Medium	28	100		

Ca(OH)_2_, calcium hydroxide; Gly, glycyrrhizin

Samples were collected at three time points: S1 (before chemomechanical preparation), S2 (after chemomechanical preparation), and S3 (after 7 days of intracanal medicament). At S3, three participants per group were lost to follow-up. In addition, 1 patient in the Ca(OH)2 group (group 1) was excluded from DNA-based RT-PCR because of insufficient DNA concentration, and 1 patient in the Gly group (group 2) was excluded from RNA-based RT-PCR due to failure of cDNA synthesis. Consequently, S3 data were available for 10 and 11 participants for DNA and 11 and 10 participants for RNA in groups 1 and 2, respectively. Participant flow is illustrated in the CONSORT flow diagram (Fig. 1).

### Diagnostic and procedural steps

The diagnostic process involved obtaining medical and dental histories, conducting clinical and radiographic examinations, and documenting the patients’ chief complaint. Clinical assessments included the detection of carious lesions, tooth discolouration, and extensive restorations. Percussion and palpation tests were performed, along with periodontal probing. Pulp necrosis was confirmed using an electric pulp tester and cold sensitivity testing. Pre-treatment radiographs were obtained with a NEW IDA dental sensor (Dabi Atlante, Brazil) to assess root morphology, periapical status, and the presence of apical radiolucency.

Each tooth was anaesthetised under strict aseptic conditions. Plaque removal and disinfection were performed using 30% hydrogen peroxide (H_2_O_2_), followed by 2.5% sodium hypochlorite (NaOCl). A sterility control sample of the tooth surface was obtained for each participant using sterile paper points. The absence of bacterial RNA in the sterility control samples was confirmed by real-time PCR. If bacterial RNA had been detected in any sterility control sample, the tooth would have been excluded from the study. Access cavities were then prepared using sterile diamond burs and irrigated with sterile saline.

### Root canal treatment and sampling

Before the first sampling (S1), working length was estimated using radiographs and the Propex IQ apex locator (Dentsply Sirona, Ballaigues, Switzerland), the root canal was filled with sterile saline solution. A sterile #15 H-file (Mani, Inc., Utsunomiya, Japan) was utilized to scrape the canal walls; circumferential filing was performed with one stroke against each canal wall to dislodge bacteria into the surrounding solution. For sample collection, three sterile paper points (Cell pack, Henry Schein, Buccinasco, Italy) were consecutively introduced to the full canal length for 60 s with a pumping action. These were then transferred into cryotubes containing RNAlater solution (Qiagen, Hilden, Germany) for subsequent DNA and RNA extraction.

Chemomechanical preparations were performed with reciprocating NiTi instruments, specifically WaveOne Gold Medium files (tip size 35, 0.06 taper; Dentsply Maillefer, Ballaigues, Switzerland). Following preparation with each instrument, canals were irrigated with 2.5% NaOCl. Upon completion of instrumentation, canals were rinsed with 5 mL of sterile saline, filled with 17% ethylenediaminetetraacetic acid (EDTA; Meta-Biomed Co. Ltd., Cheongju-si, South Korea), and activated using the EDDY sonic system (VDW, Germany), followed by another saline rinse. Subsequently, a total of 20 mL of 2.5% NaOCl per canal was delivered and activated with the EDDY system, after which NaOCl was neutralised using 5% sodium thiosulfate. A final rinse with sterile saline was performed.

The second sampling (S2) was performed immediately after chemomechanical preparation using sterile paper points. The 28 single-rooted teeth were then randomly allocated to two groups for application of the intracanal medicaments. After S2, the canals were dried and the intracanal medicaments were placed, using an amount sufficient to completely fill the root canal space to 1 mm short of the established working length. Placement was verified by gently advancing a sterile paper point to ensure adequate distribution of the medicament.

Randomisation and allocation concealment were achieved by dividing participants into the control (Ca(OH)_2_) and test (Gly) groups, with a 1:1 allocation ratio; each case was coded and sealed in sequentially numbered opaque envelopes to ensure blinding of participants. Operator blinding was not feasible due to differences in the colour and consistency of the intracanal medicaments. However, assessor blinding (laboratory personnel and the statistician) was achieved through concealed allocation and sample coding; all samples were labelled using alphanumeric codes, with no indication of the intracanal medicament used.

#### Group 1 (Ca(OH)_2_, *n* = 14)

The teeth in Group 1 received Ca(OH)_2_ (Prevest, Jammu, India), which was prepared by mixing the powder with distilled water at a 1:1 ratio and placed with a lentulo spiral (Henry Schein Inc., Melville, NY, USA).

#### Group 2 (Gly, *n* = 14)

The teeth in Group 2 received Gly, which was used in gel form at a concentration of 50 µg/mL and delivered with a plastic syringe. The Gly gel was prepared according to a previously published protocol [6]. The Gly gel was formulated in the Department of Pharmacognosy, Faculty of Pharmacy, Mansoura University, using Carbopol 940 (Lubrizol, Wickliffe, OH, USA) at a concentration of 1% as the gelling agent. The preparation was carried out under aseptic conditions, where Gly was dissolved in a small quantity of propylene glycol (50 µg/mL) before incorporation into the Carbopol dispersion. Triethanolamine was gradually added to neutralize the formulation and facilitate gel formation.

The resulting gel was monitored for pH using a calibrated pH meter and adjusted to maintain a value between 6.5 and 7.5. Each batch was visually examined to ensure homogeneity, the absence of particulate matter, and phase stability before application. The formulations were stored in sterile, airtight containers at 15–25 °C for up to 30 days. Batches showing any deviation in appearance or pH outside the specified range were discarded. These procedures ensured sterility, pH consistency, short-term stability, and overall batch quality suitable for clinical use [6].

Following placement of the medicaments, the access cavities were sealed with Cavit (ESPE, Seefeld, Germany), a temporary restorative material. No adverse effects were reported in association with the use of Gly as an intracanal medicament.

The third sampling (S3) was conducted 7 days later, after patient recall. The canals were aseptically accessed, medicaments were removed after 7 days using standardized saline irrigation with EDDY sonic activation in both groups, and samples were collected using paper points. Six patients (three from each group) did not attend the follow‑up visit; therefore, S3 samples were obtained from 22 patients.

### Molecular analysis

Bacterial samples (S1, S2, S3) from both groups were placed in cryotubes containing RNAlater solution (Qiagen) and stored at − 80 °C. Total nucleic acids were extracted using the MasterPure™ Complete DNA and RNA Purification Kit (Epicentre Technologies, Madison, WI, USA) according to the manufacturer’s instructions. The extracted nucleic acids were suspended in 35 µL of Tris-EDTA buffer (Epicentre Technologies) and divided for DNA and RNA analysis.

Additional DNase treatment was performed to eliminate contaminating DNA. The extracted RNA and DNA concentrations were measured with a NanoDrop ND 2000 spectrophotometer (Thermo Fisher Scientific, Wilmington, DE, USA). RNA samples were reverse-transcribed into cDNA using the MMLV Reverse Transcriptase First-Strand cDNA Synthesis kit (Epicentre Technologies, Madison, WI, USA) according to the manufacturer’s instructions. PCR steps were performed using sterile, nuclease-free tubes, in which the following components were combined: template RNA, free water, and Ologo(dt) Primer (10 Μm). The samples were incubated at 65 °C for 2 min in a thermal cycler with a heated lid and then chilled on ice for 1 min. For each first-strand cDNA synthesis reaction, 2 µL 10× RT reaction buffer, 2 µL 100 mM DTT, 2 µL dNTP PreMix, and 0.5 µL RiboGuard RNase were added while the PCR tubes remained on ice.

Finally, 1 µL M-MLV Reverse Transcriptase was added and the sample was gently vortexed. For reverse transcription, the reaction tubes were incubated in the thermal cycler at 37 °C for 60 min. The reaction was terminated by heating at 85 °C for 5 min. Finally, the samples were chilled on ice for 1 min. The resulting PCR product was stored at 4 °C until the next step.

In S3, one sample from Group 2 was excluded from the analysis due to cDNA synthesis failure, and one sample from Group 1 was excluded due to a low DNA concentration. Both DNA and cDNA were subjected to real-time PCR with a QuantStudio 5 Real-time PCR system (Thermo Fisher Scientific). The reaction used the following universal 16 S rRNA primers: forward primer: 5′-GAGTTTGATCCTGGCTCAG-3′ and reverse primer: 5′-GCTGCCTCCCGTAGGAGT-3′ [15].

### Quantitative PCR

A total volume of 20 µL was used for the qPCR reactions. Ten microliters of SYBR Green with low ROX (Enzynomics, Daejeon, Korea), 1 µL of forward primer (10 µM), 1 µL of reverse primer (10 µM), 6 µL of nuclease-free water, and 2 µL of either DNA or cDNA template were used in each reaction. The cycling conditions for the qPCR were as follows: initial denaturation at 95 °C for 5 min, followed by 45 cycles of denaturation at 95 °C for 20 s, annealing at 51 °C for 20 s, and extension at 72 °C for 40 s. A QuantStudio 5 Real-Time PCR System (Thermo Fisher Scientific) was used for all qPCR tests. Melting curve analysis was used to verify amplification specificity after the Ct values were calculated using QuantStudio 5 software. Negative extraction controls and no-template PCR controls were included during assay optimization and randomly throughout the experimental runs. No amplification was detected in these controls, indicating minimal risk of contamination.

The DNA and cDNA values were determined for the number of 16 S rDNA and 16 S rRNA copies per root canal sample, providing insight into bacterial load and activity. The bacterial load throughout the treatment was estimated by quantifying bacterial DNA and RNA levels. To determine unknown DNA concentrations, a standard curve was generated by serial dilution of a clinical strain of *E. faecalis*. This allowed the construction of a linear regression curve describing the association between the logarithm of DNA concentration and the Ct values obtained from the quantitative PCR. The standard curve showed linearity (R2 = 0.9898) with a slope of − 3.50. The amplification efficiency was calculated as 93.1%. The limit of detection (LOD) and limit of quantification (LOQ) were determined statistically based on the standard deviation of the residuals (S y.x = 0.4225) and the slope, resulting in an LOD of 2.5 copies/µL and an LOQ of 16.1 copies/µL [16]. The reported values represent 16 S rRNA gene copy number equivalents, not bacterial cell counts.

Bacterial samples (S1, S2, S3) from both groups were placed in cryotubes containing RNAlater solution (Qiagen) and stored at − 80 °C. Total nucleic acids were extracted using the MasterPure™ Complete DNA and RNA Purification Kit (Epicentre Technologies, Madison, WI, USA) according to the manufacturer’s instructions. The extracted nucleic acids were suspended in 35 µL of Tris-EDTA buffer (Epicentre Technologies) and divided for DNA and RNA analysis.

Additional DNase treatment was performed to eliminate contaminating DNA [15], RNA and DNA concentrations were measured with a NanoDrop ND 2000 Spectrophotometer (Thermo Fisher Scientific, Wilmington, DE, USA).

RNA samples were reverse-transcribed into cDNA using the MMLV Reverse Transcriptase First-Strand cDNA Synthesis kit (Epicentre Technologies) according to the manufacturer’s instructions. In S3, one sample from Group 2 was excluded from the analysis due to cDNA synthesis failure. The resulting cDNA was subjected to real-time polymerase chain reaction with a QuantStudio 5 Real-time PCR system (Thermo Fisher Scientific). Average DNA and cDNA values determined the number of 16 S rDNA and 16 S rRNA copies per root canal sample, providing insights into bacterial load and activity. The bacterial load throughout the treatment was estimated by quantifying bacterial DNA and RNA levels. To determine unknown DNA concentrations, a standard curve was generated by serial dilution of a clinical strain of *E. faecalis*. This allowed the construction of a linear regression curve relating the logarithm of DNA concentration to the cycle threshold (Ct) values obtained from quantitative PCR [16].

The primary end-point was defined as the between‑group difference in change in log‑transformed bacterial RNA from S2 to S3 (Δlog RNA S3–S2). The secondary end-points were as follows: (i) between‑group difference in change in log‑transformed bacterial DNA from S2 to S3 (Δlog DNA S3–S2); (ii) within‑group changes across different sampling points in log‑transformed RNA and DNA; and (iii) between‑group differences in log‑transformed RNA and DNA at each sampling point.

### Statistical analysis

Statistical analyses were performed using GraphPad Prism version 10.1.2 (GraphPad Software, San Diego, CA, USA). Continuous baseline variables (patient age data) were compared between treatment groups using independent t-tests, whereas categorical variables (sex and tooth type) were compared using the chi‑square test to assess differences in proportions between groups.

Because bacterial DNA and RNA copies exhibited right‑skewed distributions, their values were log‑transformed. Normality of the log‑transformed DNA and RNA data was assessed using the Shapiro–Wilk test, along with visual inspection using histograms and Q–Q plots. Log‑transformed bacterial DNA and RNA copies were analysed using a mixed‑effects two-way repeated‑measures analysis, with time (three sampling points: S1, S2, S3) as the within‑subject factor and treatment group (calcium hydroxide vs. glycyrrhizin) as the between‑subject factor; when the sphericity assumption was violated, the Geisser–Greenhouse correction was applied. Post-hoc pairwise comparisons were adjusted using Sidak’s correction for multiple testing. The mixed-effects repeated-measures model accommodates unbalanced data and missing observations at S3 time point under the missing-at-random assumption, allowing inclusion of all available observations to be included in the analysis.

The change in log‑transformed DNA and RNA from S2 to S3 (Δlog DNA and RNA S3–S2) was calculated for each participant and compared between groups using the Mann–Whitney U test. The Δlog DNA and RNA S3–S2 analyses included only participants with complete DNA and RNA data at both S2 and S3 (DNA: *n* = 10 and *n* = 11, and RNA: *n* = 11 and *n* = 10, in groups 1 and 2, respectively). The primary endpoint (Δlog RNA S3–S2) was tested at a two-sided significance level of 0.05. Because of the exploratory nature of the study and the limited sample size, no additional formal adjustment for multiplicity was applied across the secondary endpoints and supplementary within‑ and between‑group comparisons; accordingly, their p‑values are interpreted descriptively.

## Results

Twenty-eight participants (14 per group) were included in the statistical analysis at S1 (before chemomechanical preparation), S2 (after chemomechanical preparation). At S3 (after 7 days of intracanal medicament), data were available for 10 and 11 participants for DNA and 11 and 10 participants for RNA in groups 1 and 2, respectively (Fig. 1). No bacterial RNA was detected in any of the sterility control samples, indicating the absence of detectable tooth‑surface contamination.

Baseline comparisons showed no statistically significant differences between groups. The independent t-test demonstrated that mean patient age did not differ between Groups 1 (40.43 ± 9.19 years) and 2 (40.46 ± 9.54 years) (*p* = 0.9928). Chi-square tests indicated no significant differences in sex distribution (χ²=0.1637, *p* = 0.6857) or tooth type (χ²=0.3733, *p* = 0.5412) between the two groups.

### Mixed-effects model on log-transformed copies

Mixed-effects two-way repeated-measures analysis of the log-transformed DNA and RNA copies revealed significant main effects of time (DNA: *p* < 0.0001, Partial η^2^ = 0.968; RNA: *p* < 0.0001, Partial η^2^ = 0.967) and group (DNA: *p* = 0.0006, Partial η^2^ = 0.37; RNA: *p* < 0.0001, Partial η^2^ = 0.551). In addition, a significant group×time interaction was observed for both DNA (*p* < 0.0001, Partial η^2^ = 0.456) and RNA (*p* < 0.0001, Partial η^2^ = 0.659).

### Within-group comparison

**Fig. 2:**
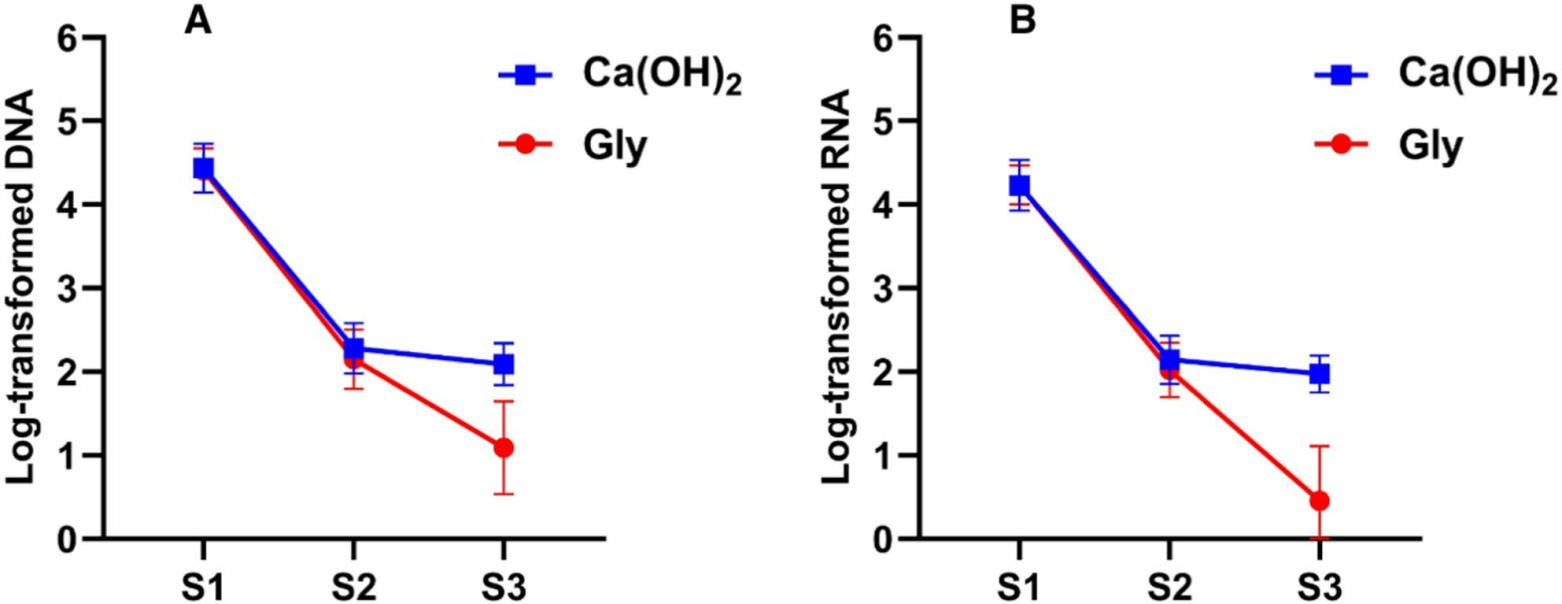
**A**,** B** Changes in log‑transformed bacterial DNA (**A**) and RNA (**B**) copy number at S1 (pre‑instrumentation), S2 (after chemomechanical preparation), and S3 (after intracanal medicament) in Group 1 (Ca(OH)_2_) and Group 2 (glycyrrhizin (Gly)). Data are presented as mean ± standard deviation

The log-transformed DNA and RNA copies across different sampling points in both groups are presented in Fig. 2. (A, B).

#### Group 1: Ca(OH)_2_

The log-transformed DNA and RNA copies at S2 and S3 were significantly lower than those at S1 (*p* < 0.0001). No significant difference in log-transformed DNA and RNA copies was observed between the S2 and S3 samples (DNA: *p* = 0.3059; RNA: *p* = 0.1139).

#### Group 2: Gly

S3 exhibited significantly fewer log-transformed DNA and RNA copies than S2 (DNA: *p* = 0.0005; RNA: *p* < 0.0001), which in turn showed significantly lower copies than S1 (*p* < 0.0001).

### Inter-group comparison

The results of the inter-group comparison across different sampling points are presented in Table 2. At S1 and S2, no significant between-group differences were observed in log-transformed DNA (S1: *p* = 0.8038; S2: *p* = 0.2957) and RNA (S1: *p* = 0.968; S2: *p* = 0.2965). However, at S3, Group 1 showed significantly higher log-transformed DNA and RNA than Group 2 (*p* < 0.0001).

**Table 2:** Inter-group comparisons of log-transformed DNA and RNA across different sampling points

		Ca(OH)_2_ group Mean ± SD (n)	Gly group Mean ± SD (n)	Effect size (Cohen’s d)	95% CI		*p*-value
					Lower	Upper	
Log-transformed DNA	S1	4.44 ± 0.29 (*n* = 14)	4.41 ± 0.26 (*n* = 14)	0.095	-0.647	0.835	0.8038
	S2	2.28 ± 0.3 (*n* = 14)	2.24 ± 0.35 (*n* = 14)	0.404	-0.349	1.15	0.2957
	S3	2.09 ± 0.25 (*n* = 10)	1.09 ± 0.55 (*n* = 11)	2.28	1.15	3.38	< 0.0001^*^
Log-transformed RNA	S1	4.23 ± 0.3 (*n* = 14)	4.23 ± 0.23 (*n* = 14)	-0.015	-0.756	0.726	0.968
	S2	2.15 ± 0.33 (*n* = 14)	1.99 ± 0.38 (*n* = 14)	0.403	-0.35	1.15	0.2965
	S3	1.98 ± 0.22 (*n* = 11)	0.46 ± 0.53 (*n* = 10)	3.16	1.83	4.46	< 0.0001^*^

^*^ Statistically significant difference at *p* < 0.05

### Δlog S3–S2

The Mann–Whitney U test demonstrated that the change in log-transformed RNA between S2 and S3 (the primary end-point) was significantly greater in Group 2 (*n* = 10) than in Group 1 (*n* = 11) [(*p* < 0.0001, effect size (rank biserial correlation) = − 0.945]. Also, the reduction in log‑transformed DNA from S2 to S3 was significantly greater in Group 2 (*n* = 11) than in Group 1 (*n* = 10) [*p* = 0.0004, effect size (rank biserial correlation) = − 0.855].

## Discussion

The persistence of viable microorganisms after root canal preparation and disinfection remains a major cause of root canal treatment failure [17]. Although chemomechanical preparation effectively reduces the microbial population in infected canals, the use of intracanal medicaments increases the likelihood of microorganism elimination from canal irregularities. These medicaments not only suppress the growth of residual strains but also help prevent recontamination of the root canal system [18].

While Ca(OH)_2_ is the most widely used intracanal medicament, its effectiveness against resistant species such as *E. faecalis* is limited. Herbal alternatives like Gly have shown promising anti-microbial properties in previous studies [5, 6]. To our knowledge, this study provides the first clinical molecular evidence of the superior antibacterial activity of Gly, supporting its potential as an effective herbal alternative in endodontic therapy.

This study focused on maxillary incisors and canines with a single root and canal, as their wide, straight anatomy permits more accurate evaluation of disinfection, minimises procedural errors, and allows better control during sample collection with paper points. In addition, the use of these teeth facilitates almost complete standardisation of instrumentation conditions and avoids anatomical complexities, such as curved canals and isthmuses, which can hinder effective disinfection. As highlighted by Smith et al. [19], achieving comparable outcomes in multi-rooted teeth is difficult owing to the increased risks of cross-contamination.

The entire clinical procedure was performed under strict rubber dam isolation, which was applied before access cavity preparation to minimize contamination of the operative field by saliva, blood, or gingival fluids, all of which may harbour bacterial microorganisms.

Root canal instrumentation was performed using WaveOne Gold files (Dentsply Maillefer) selected for their durability, flexibility, and reciprocating motion, all of which improve safety, efficiency, and effectiveness in shaping canals and removing debris [20]. NaOCl (2.5%) was employed for its strong antibacterial activity and ability to dissolve organic tissue and biofilms. Following irrigation, the canals were flushed with 5 mL of sterile saline, treated with 5 mL of 17% EDTA to remove inorganic components, and rinsed again with 5 mL of sterile saline [21, 22].

This study employed the EDDY sonic activation system to remove intracanal medicaments, leveraging its flexible polymer tip to safely navigate canals and its high-frequency oscillations to effectively eliminate debris, biofilm, and Ca(OH)_2_ residues while minimising heat generation and thermal damage. The system’s ease of use also improves irrigant penetration, resulting in enhanced root canal cleaning and disinfection [23].

Ca(OH)_2_ was selected as an intracanal medicament because of its well-established antibacterial efficacy [5], while Gly was chosen for its potent anti-inflammatory and antibacterial properties. Previous studies have demonstrated the antibacterial activity, biocompatibility, and safety of Gly at a concentration of 50 µg/mL, with no reported adverse effects, thus confirming its suitability for therapeutic use [24].

Powdered Ca(OH)_2_ was used in this study rather than ready-to-use formulations, because it is a pure material, free from additives such as barium sulphate vehicles and preservatives [25]. To minimise bias, double blinding was implemented by coding the samples so that the laboratory staff and participants remained unaware of the tested materials, thereby ensuring objective DNA and RNA detection. However, complete blinding of the operator was not possible owing to the distinct colour and consistency of the materials; this may be considered a potential limitation of the study.

To improve nucleic acid yield and concentration, canal walls were scraped with an H-file in a filing motion. three sterile paper points were then inserted and retained for 60 s with a pumping action to collect bacterial suspensions from the main pulpal area. All samples were promptly preserved in RNAlater to prevent RNA degradation.

In endodontic treatment, only viable bacteria influence treatment outcomes. This highlights the importance of targeting and eliminating active microbial populations for successful treatment [26]. Culturability and metabolic activity are widely recognised criteria for assessing bacterial viability [27].

Bacterial culturability is typically assessed through colony formation; however, under unfavourable conditions, bacteria can enter a viable but non-culturable (VBNC) state, making culturability tests insufficient [27]. RNA analysis provides an alternative method for detecting VBNC bacteria by assessing metabolic activity through RNA expression. Unlike traditional culturability tests, RNA analysis offers a more accurate evaluation of bacterial viability by reflecting ongoing metabolic processes [27, 28].

The 16 S rRNA gene is widely regarded as an ideal genetic marker in microbiology because its conserved regions permit universal primer design for broad bacterial amplification, while its variable regions enable species-specific identification. In this study, the gene was used to detect and quantify bacterial DNA within the root canal system, thereby providing insights into bacterial diversity and supporting the evaluation of the effectiveness of disinfection protocols [29].

A standard curve was used to quantitatively assess the DNA samples by comparing their signal response with that of the standard curve [30]. *E. faecalis* was selected as the reference organism for constructing the standard curve owing to its prevalence in the oral cavity of patients requiring endodontic treatment, as shown in studies employing both culture-based and molecular detection methods [30, 31].

Molecular techniques such as real-time PCR, which target bacterial DNA and RNA, allow detection and quantification of viable bacterial populations. In this study, real-time PCR was employed as the quantification method because of its high sensitivity. It can identify not only cultivable bacteria but also species that are difficult to culture or are non-cultivable [32–34].

The 50 µg/mL concentration of Gly used in this study is well below established oral safety limits. A comparable 50 µg/mL gel in a clinical trial demonstrated similar safety and strong antibacterial activity. Higher concentrations, up to 0.25%–0.5%, have also been reported as safe [35], while concentrations in the range of 12.25–50 µg/mL are considered non-cytotoxic to dental pulp stem cells [24, 36]. In addition, Gly exhibits broad-spectrum, dose-dependent inhibition of bacterial growth and biofilm formation [37, 38], which confirms its safety and effectiveness as an alternative intracanal medicament.

In both groups, the S1 samples exhibited the highest detectable levels of DNA and RNA. Following chemomechanical preparation, the S2 samples showed a significant reduction in bacterial DNA and RNA levels, highlighting the effectiveness of the treatment method. Furthermore, no significant differences were observed between the S1 and S2 samples in the different groups, likely because the same technique was consistently applied, resulting in comparable bacterial reduction. These findings are consistent with those of previous studies demonstrating the efficacy of chemomechanical preparation for reducing the bacterial load [39, 40].

In their 2007 study, Siqueira et al. [41] reported that bacterial reduction exceeded 95% in all cases following chemomechanical preparation with NaOCl as the irrigant. This finding underscores the critical role of the cleaning and shaping phase in effective root canal disinfection.

Analysis of S3 samples collected after the application of Ca(OH)_2_ as an intracanal medicament revealed no significant reduction in bacterial DNA and RNA levels compared with those in S2 samples. This suggests that although Ca(OH)_2_ possesses anti-microbial properties, its use did not significantly reduce microbial genetic material beyond the reduction already achieved by the initial chemomechanical preparation. The main bacterial reduction, therefore, occurred during the earlier treatment phase, with Ca(OH)_2_ functioning more as a stabiliser than providing a substantial additional anti-microbial effect.

In contrast, the S3 samples treated with Gly demonstrated a significant reduction in bacterial DNA and RNA levels compared with those in the S2 samples; this result indicated the enhanced anti-microbial effectiveness of Gly within the root canal. When compared with Ca(OH)_2_, Gly produced a significant decrease in microbial genetic material, highlighting its potential as a superior intracanal medicament for improved microbial control and better endodontic treatment outcomes.

The antibacterial effect of Gly is attributed to its amphiphilic structure, which disrupts bacterial membrane integrity and impairs respiration and ion transport. These alterations in membrane fatty acid composition and hydrophobicity increase permeability, promote biofilm cell detachment, and enhance overall anti-microbial activity [26].

In comparisons of Gly and Ca(OH)_2_, the latter exhibits limitations in eliminating all bacterial species, particularly those located within deep dentinal tubules or biofilms, where its high alkalinity has limited penetration [41]. Notably, *E. faecalis* can tolerate alkaline environments, contributing to its resistance. Furthermore, Ca(OH)_2_ shows restricted effectiveness in neutralising endotoxins and requires extended contact time to achieve full anti-microbial action, which may be impractical in clinical settings [41]. The greater reduction in bacterial DNA/RNA observed with Gly suggests biologically plausible potential for enhanced periapical healing; however, this remains speculative and should be confirmed in larger, long‑term clinical trials that include radiographic and clinical outcome measures as primary endpoints.

Our results corroborate the in vitro findings of Badr et al. [5] and the culture-based clinical data of Eltantawi et al. [6] by confirming the antibacterial effect of Gly. They further extend this evidence by showing, through RNA-based molecular analysis, viable bacterial that are not detectable using culture methods alone. The present results are consistent with those of previous studies [41, 42] and are further supported by the results of a systematic review [43], thereby confirming their validity. However, they differ from the findings of another study [44], likely because of variations in methodology, sample size, or experimental conditions.

The study is limited by a small sample size (*n* = 28), restriction to maxillary incisors and canines, and the inclusion of only complete cases without an intention‑to‑treat analysis, which may introduce attrition bias and limit generalizability. The inability to fully blind the operators due to differences in medicament consistency may have introduced performance bias. The molecular analysis used pooled bacterial DNA/RNA without species‑level resolution, so potential differences among specific pathogens could not be evaluated. In addition, the 7‑day follow‑up period restricted assessment of long‑term clinical effects and the clinical significance of residual post‑treatment bacteria remains uncertain, indicating a need for larger, longer‑term studies.

## Conclusions

Compared with Ca(OH)_2_, Gly demonstrated greater antibacterial activity through greater reduction in bacterial DNA and RNA levels. This may support the potential of Gly as an alternative intracanal medicament during root canal therapy.

## Data Availability

This study is part of a PhD thesis conducted at Mansoura University. The data supporting the findings of this study are available from the corresponding author upon reasonable request.

## Abbreviations

EDTA: Ethylenediaminetetraacetic acid
Gly: Glycyrrhizin
S1: First sampling (before chemomechanical preparation)
S2: Second sampling (immediately after chemomechanical preparation)
S3: Third sampling (after intracanal medicament placement)
VBNC: Viable but non-culturable
